# Regenerative Surgery with Dental Implant Rehabilitation in a Haemophiliac Patient

**DOI:** 10.1055/s-0041-1722866

**Published:** 2021-03-16

**Authors:** Christian Bacci, Alessia Cerrato, Gastone Zanette, Samantha Pasca, Ezio Zanon

**Affiliations:** 1 Section of Dentistry, Department of Neurosciences, University of Padua, Padua, Italy; 2 Department of Cardiac Thoracic and Vascular Sciences, Haemophilia Centre Unit of Coagulopathies, University Hospital of Padua, Padua, Italy

**Keywords:** dental implant, haemophilia, ridge preservation, local anaesthesia

## Abstract

This study aimed to describe the first case of regenerative surgery in haemophiliac implant. Patients with haemophilia often present dental problems. A multidisciplinary approach is suggested in case of dental surgeries to reduce the high bleeding risk. A 41-year-old male patient with mild haemophilia A (FVIII 8.4%), presenting previous epistaxis, noncomplicated tooth extractions and traumatic haemartroses, all treated with single infusions of coagulation factor concentrates, was referred to the dental clinic of the Padua University Hospital based on the recommendation of his attending dentist. At first dental visit the patient reported intense pain in the right lower second molar, with impaired chewing function. After an endodontic unsuccessful treatment the element was judged as no longer recoverable. In agreement with the patient the dental element was then extracted, after a combined administration of recombinant factor VIII 3000 IU (35 IU/kg), and tranexamic acid 1,000 mg. The extraction was performed under local anaesthesia, paraperiosteal and truncular, moderate sedation, elevation of an envelope flap. After extraction, a preservation of the alveolus was carried out with bovine matrix bone graft covered with a resorbable membrane. Three months after the surgery a flapless implant was placed after a single infusion of factor VIII 2000 IU, tranexamic acid 1,000 mg, and a local para-periostal anaesthesia, without any complication. Oral surgeon and haematologist expert in coagulation diseases must therefore collaborate together to define a shared protocol for managing surgery in those patients.

## Introduction

Patients with haemophilia (PWH) often present dental problems. Tooth extraction, minor or major surgeries are frequently performed throughout their lives. International guidelines or consensus statements have realized some recommendations to treat these patients during the oral procedures. 1 2 3 A multidisciplinary approach is suggested in case of major dental surgeries to reduce the high bleeding risk. 4 Oral surgeon and haematologist expert in coagulation diseases must therefore collaborate together to define a shared protocol for managing surgery in PWH.

Only few reports are available in literature describing implant surgery in PWH, 5 6 7 but at our best knowledge this is the first that describes a peri-implanted bone graft in this type of patients.

Here we report a case of a patient with mild haemophilia A undergoing implant-prosthetic rehabilitation, his management and outcomes.

A 41-year-old male patient with mild haemophilia A (factor VIII [FVIII] 8.4%), presenting previous epistaxis, non-complicated tooth extractions and traumatic haemarthroses, all treated with single infusions of coagulation factor concentrates, was referred to our dental clinic based on the recommendation of his attending dentist.

At first dental visit the patient reported intense pain in the right lower second molar, with impaired chewing function. The dental element had previously undergone endodontic treatment, but at this time it is still painful to percussion due to an evident osteolytic lesion at the periapical level. A second endodontic treatment was then performed, without any haemostatic covering therapy, but paying particular attention to the positioning of the hook for the rubber dam and using a particular delicacy in the endocanal instrumentation. Despite the therapy, however, the pain persisted, as did the apical imaging (radiolucency), so the element was judged as no longer recoverable.

In agreement with the patient the dental element was then extracted, after a combined administration of recombinant FVIII (lonoctocog-alfa) 3000 IU (35 IU/kg), and tranexamic acid 1000 mg intravenously 30 minutes before surgery. The extraction was performed under local anesthesia, para-periosteal and truncular, moderate sedation according to the protocol in use at our clinic, 8 and elevation of the intrasulcular mucoperiostic surgical flap, extended from the first to the third molar.

**Fig. 1:**
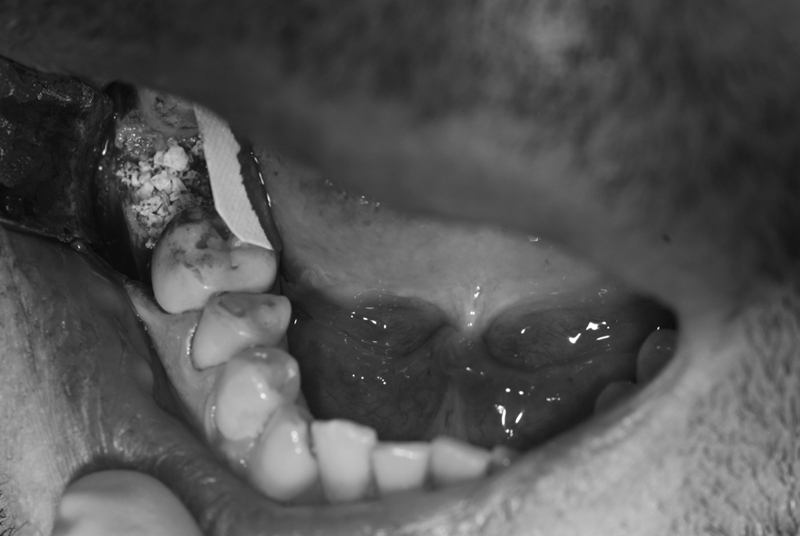
Bone grafting and the membrane during its placing.

After extraction, adequate alveolar curettage was performed, the material removed from the periapex was sent for histopathological examination and a preservation of the alveolus was performed with bovine matrix bone graft covered with a resorbable membrane ( Fig. 1 ). The surgical flap was finally sutured leaving the membrane exposed only in the occlusal surface of the post-extraction alveolus. During the intervention, no bleeding complications were recorded, with a consequent good visibility of the operating field.

The follow-up visit was performed 3 weeks after extraction, with the histological examination report that left for the diagnosis of apical cyst.

Three months after the surgery, an intraoral X-ray was taken to verify the bone healing and integration of the graft. The mucosa appeared normochromic and normotrophic, so an endosteal implant with flapless technique was placed after a single infusion of lonoctocog-alfa 2,000 IU, tranexamic acid 1000 mg and a local para-periostal anesthesia.

**Fig. 2:**
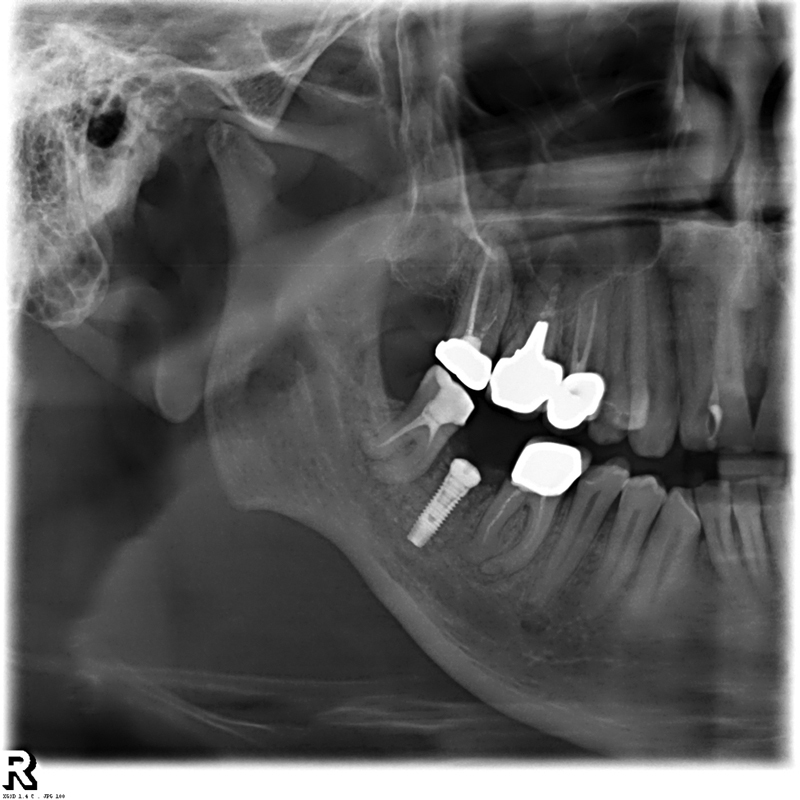
Half-panoramic X-ray showing the correct endosseous implant position and perfect integration of the bone graft.

Access to the underlying bone tissue was obtained by means of a circular mucotome. After bone preparation, a 4 × 11 titanium implant and a transmucosal healing screw were directly inserted, to avoid a second surgical access for the uncovering of the implant head ( Fig. 2 ). After the surgery, a new panoramic X-ray was taken to verify correct implant positioning.

The patient was prescribed local antiseptic therapy, antibiotic therapy and analgesic to avoid possible surgical site infections and reduce the pain.

Following adequate anti-bleeding coverage and a well-defined surgical approach, the patient did not present any bleeding events, neither during surgery, nor during implant placement, nor during the follow-up period.

There was no need to administer any post-procedure factors or other haemostatic agents and there was no need for additional surgical maneuvers.

Due to the bleeding complications that haemophilic patients may incur, they do not find easy access to dental care, except for minimally invasive procedures, which do not involve a risk of bleeding, and in adequately equipped hospital facilities, in which there is close collaboration between the coagulation disorder expert and odontologist. In this way too many times the haemophilic patient does not receive an excellent standard of treatment which instead he would have the right to be able to benefit in perfect safety. In this case, we have applied our validated protocol for dental extractions. 9

The intervention was performed in conscious sedation to reduce the discomfort associated with the procedure and allow for better homeostasis to be maintained. 10

Although it is only a case report, this article describes how with a correct multidisciplinary approach and management of the haemophilic patient, the adverse events can be avoided by allowing him to access dental treatments of the highest standard.

## References

[1] Laino L Cicciù M Fiorillo L Surgical risk on patients with coagulopathies: guidelines on hemophiliac patients for oro-maxillofacial surgery Int J Environ Res Public Health 2019 16 08 1386 10.3390/ijerph16081386 PMC6518229 30999657

[2] Hewson I D Daly J Hallett K B Consensus statement by hospital based dentists providing dental treatment for patients with inherited bleeding disorders Aust Dent J 2011 56 02 221 226 21623817 10.1111/j.1834-7819.2011.01328.x

[3] Brewer A Correa M E Guidelines for dental treatment of patients with inherited bleeding disorders World Federation of Hemophilia Treatment of Hemophilia Monograph 2006. Accessed May 2020 at:http://www.wfh.org

[4] Escobar M A Brewer A Caviglia H Recommendations on multidisciplinary management of elective surgery in people with haemophilia Haemophilia 2018 24 05 693 702 29944195 10.1111/hae.13549

[5] Gornitsky M Hammouda W Rosen H Rehabilitation of a hemophiliac with implants: a medical perspective and case report J Oral Maxillofac Surg 2005 63 05 592 597 15883931 10.1016/j.joms.2005.01.009

[6] Calvo-Guirado J L Romanos G E Delgado-Ruiz R A Infected tooth extraction, bone grafting, immediate implant placement and immediate temporary crown insertion in a patient with severe type-B hemophilia BMJ Case Rep 2019 12 03 e229204 10.1136/bcr-2019-229204 PMC6510142 30904898

[7] Rosen H Gornitsky M Cementable implant-supported prosthesis, serial extraction, and serial implant installation: case report Implant Dent 2004 13 04 322 327 15591993 10.1097/01.id.0000144511.43654.d3

[8] Manani G Bacci C Zanette G Facco E Contemporary state of sedation in dentistry Dent Cadmos 2012 80 07 357 369

[9] Zanon E Martinelli F Bacci C Zerbinati P Girolami A Proposal of a standard approach to dental extraction in haemophilia patients. A case-control study with good results Haemophilia 2000 6 05 533 536 11012698 10.1046/j.1365-2516.2000.00423.x

[10] Gupta A Epstein J B Cabay R J Bleeding disorders of importance in dental care and related patient management J Can Dent Assoc 2007 73 01 77 83 17295950

